# Spatiotemporal analyses of neural lineages after embryonic and postnatal progenitor targeting combining different reporters

**DOI:** 10.3389/fnins.2015.00087

**Published:** 2015-03-17

**Authors:** Maria Figueres-Oñate, Jorge García-Marqués, Maria Pedraza, Juan Andrés De Carlos, Laura López-Mascaraque

**Affiliations:** Instituto Cajal-Consejo Superior de Investigaciones Científicas, Department of Molecular, Cellular and Developmental NeurobiologyMadrid, Spain

**Keywords:** neurogenesis, gliogenesis, cell fate, cell division, electroporation, neurons, glial cells

## Abstract

Genetic lineage tracing with electroporation is one of the most powerful techniques to target neural progenitor cells and their progeny. However, the spatiotemporal relationship between neural progenitors and their final phenotype remain poorly understood. One critical factor to analyze the cell fate of progeny is reporter integration into the genome of transfected cells. To address this issue, we performed postnatal and *in utero* co-electroporations of different fluorescent reporters to label, in both cerebral cortex and olfactory bulb, the progeny of subventricular zone neural progenitors. By comparing fluorescent reporter expression in the adult cell progeny, we show a differential expression pattern within the same cell lineage, depending on electroporation stage and cell identity. Further, while neuronal lineages arise from many progenitors in proliferative zones after few divisions, glial lineages come from fewer progenitors that accomplish many cell divisions. Together, these data provide a useful guide to select a strategy to track the cell fate of a specific cell population and to address whether a different proliferative origin might be correlated with functional heterogeneity.

## Introduction

Neural cells are originated from a complex mix of distinct progenitors, which are pluripotent stem cells with restricted differentiation potential. The first progenitor cells are a subset of non-committed neuroectodermal progenitors that become specified as neural progenitor cells (NPCs). Subsequently, NPCs undergo repeated symmetric divisions to self-renew while they divide asymmetrically to produce distinct neural and glial lineages at different time points. Those progenitors should maintain a balance between self-renewal and lineage commitment, becoming either neural stem cells or committed progenitors that undergo a limited number of divisions producing a lineage-restricted cell progeny. After an initial symmetrical amplification of the progenitor pool, radial glial cells (RGC) start to undergo asymmetrical divisions producing more restricted progenitors cells, including intermediate progenitor cells (see reviews by Götz and Huttner, 2005; Franco and Müller, 2013). RGCs differentiate at early developmental stages into neuron-committed progenitor cells and at later stages into glial-restricted precursors (Noctor et al., 2002; Anthony et al., 2004). Perinatally, some RGCs continue generating neurons and oligodendrocytes, while others RGCs transform into adult subventricular zone (SVZ) astrocytes, that differentiate to adult neural stem cells (Kriegstein and Alvarez-Buylla, 2009). However, the precise relationship between neural progenitors and their commitment to each cell lineage is still largely unknown. Recent data indicates a role for the transcription factor Pax6 in regulating the orientation of cleavage plane that is crucial for symmetric vs. asymmetric cell divisions (Asami et al., 2011).

During NPCs life, it is difficult to differentiate between the clonal contribution of a particular cell in development, and its ongoing contribution in the adult. So far, several strategies for cell lineage analyses trace the development and cell progeny of NPCs. Different approaches and transgenic mice have been used to analyze either the cell division pattern or the progenitor cycle progression as FUCCI reporters (Sakaue-Sawano et al., 2008). Moreover, newly generated cells can be labeled by birth-dating methods, such as retrovirus infections, incorporation of thymidine analogs and transgenic mice (Imayoshi et al., 2011). Further, genetic and fate-mapping studies revealed the temporal and spatial origin of different neuronal populations. However, most of these approaches have technical limitations (Blanpain and Simons, 2013). Before attempting to interpret NPCs commitment, it is necessary to distinguish the fate of daughter cells or even to track, over long time periods, the progeny of a single cell. Thus, the lineage tracer should be retained permanently and transmitted to all progeny of the founder cell, and should not be spread to unrelated cells (Kretzschmar and Watt, 2012). DNA-mediated gene transfer by electroporation allows the permanent labeling of a particular neural cell population and its progeny. Lineage tracing with multicolor reporters can provide information on cell tracking, cell viability, cell function, and cell proliferation. However, depending on the selected gene vectors to clone the DNA fragments, transfected plasmids may be episomally transcribed or may be designed to be integrated into the host cell genome (Yoshida et al., 2010) allowing a long-term stable expression. Integration of reporter proteins in neural progenitors genome by co-electroporation of transposon vectors (Ding et al., 2005; Nakanishi et al., 2010), generates a specific and permanent color combination by random transposition, allowing the analyses of *in vivo* single cell progeny (García-Marqués and López-Mascaraque, 2012; García-Moreno et al., 2014; Loulier et al., 2014; Siddiqi et al., 2014).

Here, we undertake a comparative analysis of the labeled adult cell progeny by targeting neural progenitors after embryonic and postnatal co-electroporation of integrable and non-integrable constructs. Our results showed that the percentages of labeled cell types differed greatly depending on the construct. We highlighted the importance of the selected strategy to efficiently track the whole and specific cell progeny of neural progenitors.

## Materials and methods

### Animals

Wild type C57BL/6 mice of either sex were raised at the Cajal Institute animal facility. All of the experiments were performed according to ethical regulations on the use and welfare of experimental animals of the European Union (2003/65/CE) and the Spanish Ministry of Agriculture (RD 1201/2005 and L 32/2007). Bioethical committee of CSIC approved these experimental procedures. Day of vaginal plug detection was defined as the first embryonic day 0 (E0) and the day of birth was defined as postnatal day 0 (P0).

### Tissue processing

Mice were analyzed in both embryonic and adult stages (considering adult stages from P30 onwards). Pups from P1 to P6 were anesthetized by hypothermia, whereas mice from P6 onwards were anesthetized with intraperitoneal injection of pentobarbital (40–50 mg/Kg body weight), and then perfused with 4% paraformaldehyde (PF). Brains were postfixed in PF overnight, and coronal or sagittal sections (50–100 μm) were serially obtained with a vibratome.

### Expression vectors

**Figure 1:**
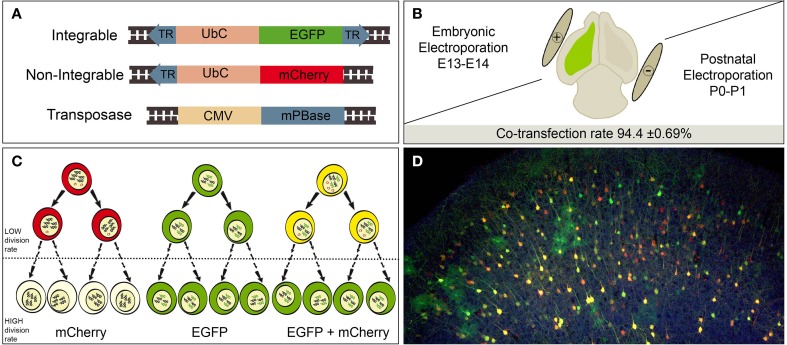
**Experimental procedure. (A)** Simplified diagram of the designed constructs. Integrable construct contains an EGFP (enhanced green fluorescent protein) encoding region under the Ubiquitin C human gene (UbC) promoter. The transposase recognizes the terminal repeats sequences (TR, blue), cleaves and integrate flanked region into the host cell genome. Non-integrable construct encodes mCherry fluorescent protein under UbC promoter. Construct modified by removing the 3′ TR from the original vector. PiggyBac transposase (mPBase) under the control of the ubiquitous CMV promoter. **(B)** A mixture of the three constructs was injected (green color) and electroporated into the LV at either postnatal or embryonic stages. Positive and negative poles of the tweezer electrode were orientated to target the ventricular dorso-lateral area. The co-transfection rate of integrable and non-integrable constructs 2 dpi was 94.4 ± 0.69%. **(C)** Transfected cells expressed red, green or yellow fluorescence depending of reporter uptake (one or both reporters). Red expression will be lost by dilution of non-integrable plasmid in the recurrent dividing cells. Consequently, green integrable vector will be the unique label after several rounds of divisions. Dotted line means undetermined number of cell divisions. **(D)** Adult cortical labeled cells after embryonic co-electroporation of the three constructs, showing red, green and yellow cells.

We used the piggyBac transposon integrable plasmid, pPB-UbC-EGFP, encoding the enhanced green fluorescent protein (EGFP) under the human ubiquitous Ubiquitin C (UbC) promoter, provided by Prof. Bradley (Yusa et al., 2009). This plasmid is flanked by two terminal repeats sequences (TRs) recognized by the piggyBac transposase (mPBase). The mPBase inserts the vector, including the reporter gene, directly into the genome of the transfected cell at TTAA repeat regions (Figure 1A, integrable). This allows the analyses of the entire cell progeny regardless of its mitotic activity. We also designed a new plasmid encoding mCherry fluorescent protein under the UbC promoter, using pPB-UbC-EGFP as the cloning vector. The region containing the EGFP and the 3′TRs was excised using the enzymes *BamHI/MluI*, to avoid the genomic insertion of the mPBase. Next, the region encoding mCherry protein, amplified from the StarTrack pPB-GFAP-mCherry (García-Marqués and López-Mascaraque, 2012), was cloned into the excised vector in the *BamHI/MluI* sites. Thereby, we generated a new non-integrable construct (Figure 1A, non-integrable) that will remain as episomal plasmid in the electroporated cells.

### *In utero* electroporation (IUE)

**Figure 2:**
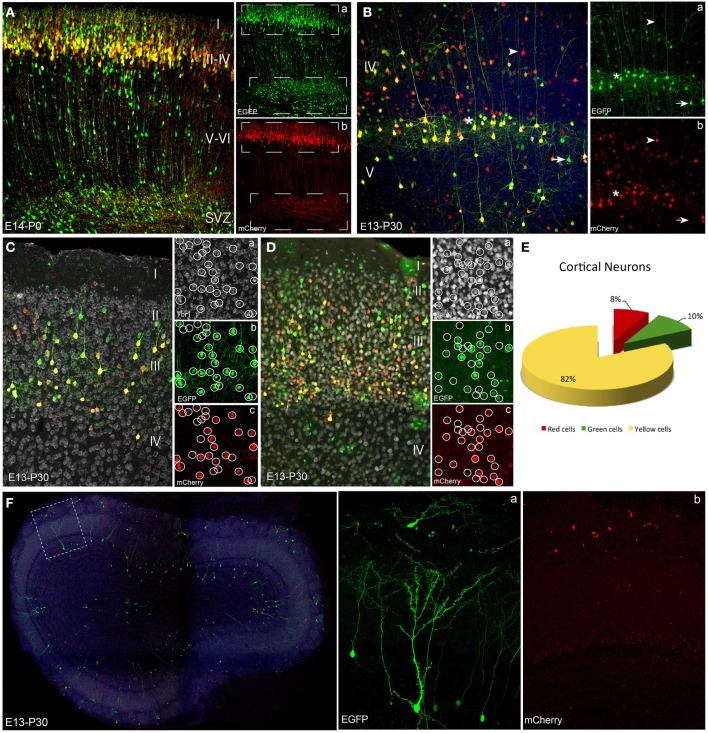
**Neuronal lineage after embryonic electroporation. (A)** Cortical P0 cells transfected at E14. Neurons in layers II and IV expressed interchangeably red or green plasmids (upper box in **a,b**). Cells located close to subventricular zone lost the mCherry non-integrable vector. This proliferative zone mostly expressed the integrated plasmid (EGFP), because the mCherry-plasmid dilution through cell division rounds (lower box in **a,b**). **(B)** Adult (P30) cortical cells labeled after IUE at E13 expressed either green (arrow) or red (arrowhead) reporters. Yellow cells (asterisk) co-expressed both constructs. At adult stages non-integrable constructs maintained a stable cell expression. **(C,D)** IUE performed at E13 and analyzed at adult stages (P30). Tbr1- (**Ca**) and NeuN-positive cells (**Da**) revealed the neuronal fate of cortical neurons expressed both the integrated (green, **b**) and non-integrated (red, **c**) constructs. **(E)** Percentage of adult cortical neurons (IUE at E14, analysis P30, *n* = 4 animals, 4 sections per animal) expressing green- (9.6 ± 1.5%), red- (8.1 ± 1.5%) or both constructs (82.3 ± 1.6%). **(F)** Interneurons in the olfactory bulb after IUE at E13. Olfactory bulb transfected interneurons expressed only the green integrated construct **(a)**. Separate confocal channels demonstrate the lack of mCherry signal **(b)**. IUE: *In utero* electroporation.

IUE was performed as previously described (García-Marqués et al., 2014). Briefly, E13-E14 pregnant mice were anesthetized with isofluorane (Isova vet, Centauro), and placed in a thermic plate. The skin and the abdominal cavity were cut, opened, and the uterine horns exposed. The DNA mixture solution consisted of an equal amount of pPB-UbC-EGFP and UbC-mCherry plasmids, plus half the amount of mPBase and 0.1% Fast Green. The final concentration was 1 μg/μL, and 1 μl of the solution was injected into the lateral ventricle of each embryo by a pulled glass micropipette. After that, the head of each embryo was placed between 3 mm tweezer-type electrodes (Sonidel) and 5 electric pulses of 50 ms length were passed after 950 ms intervals using an electroporator. In all cases, the electroporated region was the ventricular zone in the dorso-lateral area (Figure 2B). The voltage varied depending on the embryonic day from 33 V at E13 to 35 V at E14. After electroporation, the uterus was repositioned and the abdominal cavity was sutured. Pregnant mice received a subcutaneous injection of both 5 mg/kg of the antibiotic enrofloxacine (Baytril; Bayer, Kiel, DE) and 300 μg/kg of the anti-inflammatory/analgesic meloxicam (Metacam; Boehringer Ingelheim) and embryos were allowed to continue developing until desired.

### Postnatal electroporation

Pups (P0/P1) were anesthetized by hypothermia and trans-illuminated under a cold light to facilitate the visualization of the lateral ventricles. The ventricular cavity was injected with 2 μl of the plasmid mix, and 5 electric pulses of 100 V were applied (50 ms each pulse, separated by 950 ms intervals). Electroconductive LEM Gel (DRV1800, MORETTI S.P.A.) was placed in both electrode paddles to avoid pups damage and get a successful current flow. The positive electrode was positioned to direct the negatively charged DNA. After the pulses, animals were reanimated and returned to the mother.

### Immunohistochemistry

Electroporated labeled cells were identified by their morphological features and then confirmed by the expression of different molecular markers. The following primary antibodies were used: rabbit anti-Tbr1 (1:500, Chemicon AB9616) and mouse anti-NeuN (1:500, Chemicon MAB377) to classify neuronal cells; rabbit anti-PDGFRα (1:300, SantaCruz-338), mouse anti-S100β (1:300), rabbit anti-Olig2 (1:3000, Millipore), rat anti-MBP (1:1000, Abcam R7349) to identify glial lineages. In all cases the secondary antibodies were labeled with an infrared fluorochrome (1:1000, Alexa Fluor 633 or 647, Molecular Probes).

### Image processing

Fluorescent labeling was visualized using an epifluorescence microscope (Nikon, Eclipse E600) with the appropriate filter cubes: rhodamine (569–610 nm) and fluorescein (450–490 nm), to visualize mCherry and EGFP, respectively. Then, images were obtained on a Leica TCS-SP5 confocal microscope. Maximum projection images were created using confocal software (Leica LAS AF Software). Captured images were processed to equally adjust the contrast using Adobe Photoshop CS5 software.

### Statistical analysis

For quantification four coronal sections from *n* = 4 mice per age were imaged. Co-transfection efficiency, of both red and green fluorescent proteins, was calculated through the LAS AF software (Leica) in electroporated animals (IUE at E14), at 2 days post-IUE. Quantitative analysis of the expression of integrable and non-integrable constructs in the different neural populations was performed by manual selection of positive cells labeled by one or both constructs in the different neural populations. To determine the intensity for each fluorescent protein in every selected cell, data were obtained using customized routine designed in Matlab (The Math Works Inc., USA). Quantification of the number of labeled cells was performed in mice electroporated at embryonic (E14) and postnatal stages (P1). Short-term group was sacrificed 5 days after electroporation (P6) and the long-term group 1 month after (P30). To determine the statistical significance, One-Way analysis of variance (ANOVA) was performed by SigmaPlot 13 (Systat Software), and values were represented as the mean ± SEM. *Post-hoc* analysis was made by pairwise comparison procedures by Turkey's significance test. A confidence interval of 95% (*p* < 0.05) was required to considered values statistically significant.

## Results

### Neuronal progeny after *in utero* electroporation of genome-integrable (EGFP) vs. non-integrable (mCherry) plasmids

We used the piggyBac system to genetically label progenitor cells and their progeny with reporter fluorescent proteins under the ubiquitous UbC promoter. We compared labeled neural progeny after *in vivo* co-electroporation of the following three vectors (Figure 1A): (1) Integrable construct, encoding the green fluorescent protein (EGFP) flanked by transposase recognition sites (Terminal repeats, TRs), (2) Non-integrable construct encoding the fluorescent protein red fluorescent protein (mCherry) without TRs in 3′ region, and (3) the transposase of the piggyBac system (mPBase). In all cases, these three constructs were injected and co-electroporated targeting the dorso-lateral ventricular zone, at either embryonic or postnatal ages (Figure 1B). Cell progeny showed three different patterns of reporter protein expression: mCherry positive cells (red), EGFP positive cells (green) and cells expressing both reporters (yellow), depending on the uptake of one or both constructs into progenitors during electroporation. Red positive cells (expressing non-integrated sequence) will lose their label after multiple rounds of cell division, as a result of the dilution of the construct. Thus, the red non-integrable construct will not allow tracking the whole cell progeny. Red plasmid dilution will also occur in the case of yellow positive cells expressing both fluorescent reporters. In that case cells coming from yellow progenitors after divisions will only express the green integrated reporter (Figure 1C). On the other hand, green positive cells will maintain the label through all cell division rounds, allowing the complete cell progeny tracking. These three color-patterns were observed *in vivo* in electroporated cells as shown in Figure 1D. To precisely determine cell dilution by proliferation, we performed *in utero* co-electroporation (IUE) at different embryonic stages, with a co-transfection rate of 94.4 ± 0.69% (*n* = 4 mice, 4 slices per brain). At perinatal stages, cells transfected into the LV at E14, produced a large number of labeled neuronal cells (Figure 2A). Decomposition in separate channels (red and green, Figures 2Aa,b) clearly revealed that neurons located in upper layers expressed both the integrated and non-integrated sequences (upper boxes in Figures 2Aa,b). However, the majority of cells located close to the SVZ, as well as those cells ascending to cortical layers, only expressed the integrated green reporter (Figures 2Aa,b, lower boxes).

To examine whether these embryonic progenitor cells exhibited distinct cell fate potentials over time, the cell progeny analyses were also performed at mature cortical stages. At P30, cells transfected by IUE at E13 retained the expression of both reporters (Figure 2B): EGFP (integrated, Figure 2Ba) and mCherry (non-integrated, Figure 2Bb). Therefore, layer V cortical neurons exhibited red, green and yellow (combination of both reporters) fluorescence, indicating that the expression of non-integrated sequences remained stable over time in cells generated after a limited number of progenitor divisions. Immunohistochemical analysis was performed using the neuronal marker Tbr1 (T-box brain gene 1), expressed in adult pallial neurons of upper- and lower- cortical layers (Figures 2Ca–c), as well as the neuronal nuclei marker, NeuN (Figures 2Da–c). Both markers co-localized with green, red and yellow transfected cells verifying the neuronal lineage. To quantify the percentage of cells expressing one or both constructs we analyzed only those mice with IUE at E14 (*n* = 4, and 4 representative sections per animal), to avoid the dilution effect within the neuronal cortical layers. Analysis of the adult labeled cells showed that 82.3 ± 1.6% of the neuronal population expressed both constructs, 9.6 ± 1.5% expressed just the green while the red construct was only expressed in the 8.1 ± 1.5% of neurons (Figure 2E).

Additionally, after IUE of integrable and non-integrable plasmids in the dorso-lateral part of the embryonic ventricular zone, labeled cells were located in the adult olfactory bulb (OB), corresponding to interneurons originated at perinatal stages. Those labeled periglomerular and granule cells only expressed the green reporter protein (Figure 2F). The unique presence of green positive cells (Figure 2Fa), but not red cells (Figure 2Fb), clearly indicated that those SVZ progenitors underwent repeated divisions during embryonic stages before being differentiated into OB interneurons.

### Expression of reporter genes in neuronal committed-lineages after postnatal electroporation of genome-integrable (EGFP) vs. non-integrable (mCherry) plasmids

**Figure 3:**
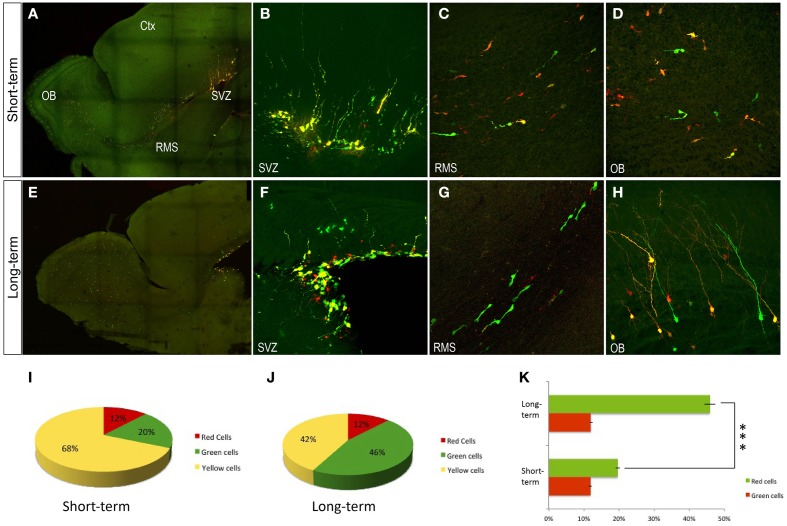
**Olfactory bulb interneurons after postnatal electroporation (P1). (A–D)** P6 transfected cells expressed the integrated and non-integrated constructs interchangeably. **(B)** In the SVZ, transfected red and green cells showed glial morphology. **(C)** In the rostral migratory stream large cells labeled were directed to the olfactory bulb. **(D)** OB displayed a huge number of neuroblasts in the ependymal zone. **(E–H)** Adult transfected cells. **(F)** In the SVZ glial-like cells co-expressed both plasmids. **(G)** In the RMS migrating neuroblast mostly expressed just the EGFP plasmid (integrated). **(H)** Transfected OB cells co-expressed both constructs. Sagittal sections. **(I–K)** Quantitative analyses after postnatal electroporation (P1). **(I)** Short-term analyses after postnatal electroporation (P1 to P6) showed that OB interneurons mostly expressed both constructs: yellow cells, 68.5 ± 1.5%; green cells, 19.6 ± 4.3% and red cells, 11.9 ± 5%. **(J)** Long term survival times after postnatal electroporation (P1 to P30) showed the following distribution: yellow cells, 42.1 ± 4.6%; green cells, 45.9 ± 3.3% and red cells, 12 ± 3%. **(K)** Increment of red cells in short term analyses, One-Way ANOVA showed a high statistical significance lower that *P* < 0.001 Ctx, cortex; OB, Olfactory bulb; RMS, rostral migratory stream; SVZ, subventricular Zone.

To further individually explore the postnatal progeny of precursor cells, co-electroporation of the plasmid mixture was performed into the neonatal SVZ (Figure 3). Short-term expression analysis (P6, Figures 3A–D,I) revealed an equivalent expression of both integrable and non-integrable reporters. Labeled cells corresponding to either progenitor cells remained within the SVZ (Figure 3B), neuroblasts along the rostral migratory stream (RMS, Figure 3C) or incoming neuroblasts and immature interneurons within the OB (Figure 3D). Those interneurons mostly expressed both constructs after short-term survival times (yellow cells: 68.5 ± 1.5%). Equivalent expressions of both integrable (green cells: 19.6 ± 4.3%) and non-integrable (red cells: 11.9 ± 5%) reporters were determined after quantification analysis (Figure 3I). There were not significant differences between the percentage of labeled green and red cells after One-Way ANOVA analyses. Different expression pattern was observed at long-term survival analysis (P30, Figures 3E–H,J). In the SVZ, glial-like cells strongly co-expressed both plasmids (Figure 3F), suggesting that they did not undergo enough division rounds to lose the non-integrable plasmids. Cell expression of non-integrable (red) plasmids decreased along the RMS cells (Figure 3G) that mostly expressed the integrable EGFP plasmid. Besides, OB interneurons co-expressed both plasmids (Figures 3H,J; yellow cells: 42.1 ± 4.6%), but since in the RMS the neuroblasts were green labeled, those red positive cells in the OB probably corresponded to the first incoming OB interneurons. This was corroborated after the One-Way ANOVA analysis, showing a significant increment (*P* < 0.001) in the percentage of green OB interneurons in long-term experiments (Figure 3K). By contrast there were not significant differences in the percentage of red cells comparing short- and long-term experiments. Thus, those data provided insights into cell fate determination analyses of SVZ progenitor cells.

### Tracking glial cell progeny after electroporation of genome-integrable (EGFP) vs. non-integrable (mCherry) plasmids

**Figure 4:**
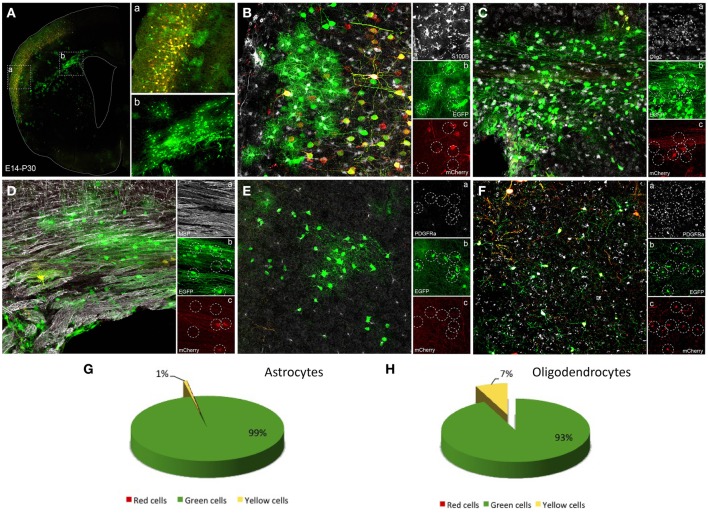
**Glial cells labeled after E14 *in utero* electroporation. (A)** Coronal section showing a general view of adult transfected cortical cells. (**a**) High magnification of box a showing cortical neurons expressing both constructs while astroglial cells just expressed the integrable construct (green). (**b**) High magnification of lower cortical layers and white matter showing glial cells labeled with green reporter protein corresponding to integrable vector. **(B–F)** Immunohistochemistry to identify the phenotype of transfected labeled cells. **(B)** S100β positive cells **(a)** co-localized only with green transfected cells (integrable construct, **b**), but never with the non-integrable vector (red, **c**). **(C)** Olig2 positive cells **(a)** mostly colocalized with cells expressed the green reporter (integrable construct, **b**) whereas some olig2 positive cells co-expressed the non-integrable construct **(c)**. **(D)** MBP positive cells **(a)** colocalized with cells mostly expressing the integrable construct **(b)**, although some red cells were found **(c)**. **(E)** PDGFRα-positive cells (**a**, NG2 cells) colocalized with transfected cells expressing only the green integrated construct **(b,c)**. **(F)** PDGFRα positive cells **(a)** expressing the integrable and the non-integrable constructs (**b,c**). **(G)** Percentage of adult astrocytes (IUE at E14, analysis P30, *n* = 4 mice, 4 sections per animal) expressing green- (99.13 ± 0.19%) or both constructs (0.8 ± 0.19%). **(H)** 93 ± 3.9% of the adult oligodendroglial cells after IUE at E14 expressed just the green construct while 7 ± 3.9% expressed both constructs.

After embryonic mixture co-electroporation, adult cortical neurons showed an equal expression of both plasmids (Figure 4A), whereas glial cells expressed predominantly the green plasmid. Those glial cells were located through upper cortical layers (Figure 4Aa), lower cortical layers and within the corpus callosum (Figure 4Ab). Those cells expressing just the green reporter protein (Figures 4Bb,c) of the integrable construct correspond to mature astrocytes since they co-expressed the S100β marker (Figure 4Ba). Thus, ventricular embryonic progenitors underwent a large number of divisions to produce those astrocytes. However, although most oligodendrocytes expressed the green fluorescent protein (integrated construct, Figures 4Cb, Db) some located within the white matter expressed also the non-integrable red construct (Figures 4Cc,Dc). Oligodendrocytic fate of labeled cells was detected by the oligodendrocyte transcription factor, Olig2, and myelin basic protein, MBP (Figures 4Ca,Da). PDGFRα positive cells (Figures 4Ea, Fa) mainly co-expressed the green integrable construct (Figure 4Eb,c), although in some cases both constructs were found in groups of PDGFRα positive cells (Figures 4Fb,c). Quantitative analysis of the expression of one or both reporter fluorescent proteins was performed separately in both astroglial and oligodendroglial populations. Only cells that displayed typical morphology were included into the analysis. Quantification of adult astrocytes percentage, after IUE at E14, revealed that 99.13 ± 0.19% expressed only the green construct. A low percentage of yellow astrocytes (0.8 ± 0.19%) were co-labeled (Figure 4G). No sections with astrocytes expressing only the red non-integrable constructs were found. Quantification of percentage of adult oligodendrocytes, IUE transfected at E14, showed that 93 ± 3.9% expressed just the green construct while 7 ± 3.9% expressed both green integrable and red non-integrable constructs (Figure 4H). No red expressing cells were found.

**Figure 5:**
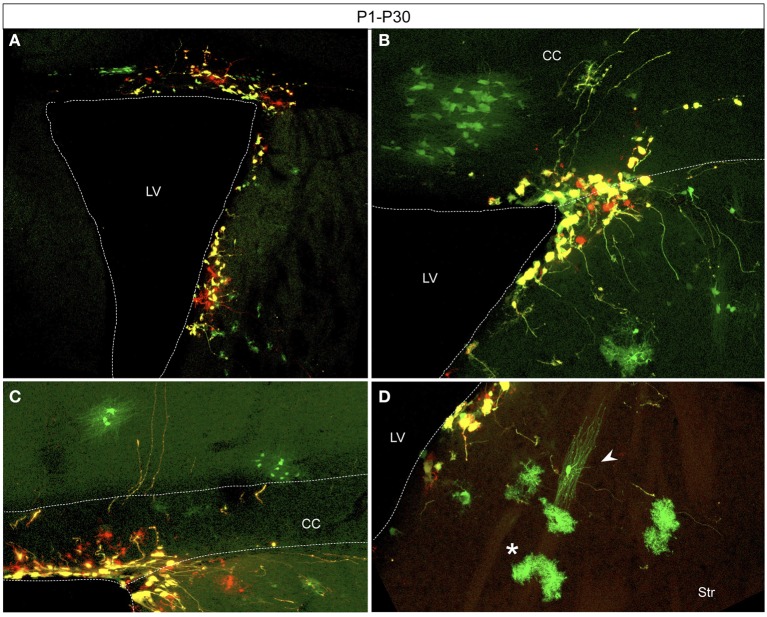
**Adult glial transfected cells after postnatal electroporation (P1) (A)** Electroporation area showing targeted cells located in the dorso-lateral wall of the lateral ventricle. **(B–D)** Glial cells derived from SVZ progenitors expressed just the integrated construct (green) whereas remaining SVZ-cells highly co-expressed both constructs (yellow). Transfected cells with glial morphology located in the withe matter **(B)**, in lower cortical layers **(C)**, and in the striatum **(D)**. Typical morphologies of astrocytes (**D**, asterisk) and oligodendrocytes (**D**, arrowhead). CC, corpus callosum; LV, Lateral ventricle; Str, striatum.

Electroporation of postnatal SVZ progenitors yielded glial cells after several weeks (Figure 5). Interestingly, those glial cells, generated from postnatal precursors, expressed only the integrable construct at P30. After postnatal electroporation into the dorsolateral part of the neonatal SVZ (Figure 5A), glial cells were found in the white matter (Figure 5B) and within the gray matter both in lower cortical layers (Figure 5C) and the striatum (Figure 5D). Consequently those glial cells probably arose from SVZ progenitors after a long number of divisions.

## Discussion

This work addresses, characterizes and compares the labeled adult cell progeny after postnatal and *in utero* electroporation of different fluorescent reporters. Long-term labeled cells were obtained by the use of the piggyBac transposon system in both cerebral cortex and SVZ-RMS-OB pathway, at embryonic and postnatal mice stages. This provides a useful guide to tracking the cell fate of a specific cell population.

### Importance of the reporter gene integration into the host genome

The use of piggyBac transposon system ensured the tracking of glial cell populations, which would not be possible without plasmid integration into the genome of transfected cells (García-Marqués and López-Mascaraque, 2012; García-Marqués et al., 2014). Otherwise, the non-integrable fluorescent reporter gene will be lost in successive cell divisions, and the fluorescence will be unequally inherited between daughter cells. Moreover, one of the main challenges faces by *in vivo* gene electroporation is the presence of non-integrable plasmids that remain in an episomal form (Sato et al., 2007). This is of particular relevance to track glial cell lineages or neuronal cells differentiated after a large number of divisions in the adult brain. Our findings indicated that transposable constructs (piggyBac transposon system) are required to achieve the complete integration into the host cell genome, allowing to track those lineages generated after several rounds of divisions. Transfected progenitor cells with an integrable plasmid maintained long-term stable fluorescent reporter expression, while cells containing non-integrable plasmids lost their expression throughout sequential dilution of the reporter by successive cell divisions. Non-integrable plasmids will be only maintained in those cells with a reduced number of divisions. Accordingly, transposon systems have been used to avoid the plasmid dilution and to track all the cell progeny (Yoshida et al., 2010; Chen and LoTurco, 2012; Kita et al., 2013).

### Biological implication of non-integrable plasmids

We evidenced the stable expression of non-integrable plasmids in differentiated cells, several months post-electroporation. Non-integrable plasmids were actively expressed in those cells, as previously reported in RNAi and gene expression analyses (Matsuda and Cepko, 2007; LoTurco et al., 2009). Moreover, the expression of non-integrable genomic constructs persisted in cells generated after a reduced number of divisions. This fact became interesting when the requirement is to label the first cell generation after overexpression/downregulation cell analyses. However, the presence of non-integrable reporter proteins will be negative for other studies such as clonal multicolor analysis from a single cell (García-Moreno et al., 2014; Loulier et al., 2014; Siddiqi et al., 2014). Those color-clonal methods are based on the genomic integration of stochastic combination of fluorescent proteins in each progenitor, maintained throughout the whole progeny. The permanent expression of non-integrated reporter fluorescent proteins in cells generated after few cell divisions will interfere with the specific code of colors of clonally related cells. However, those non-integrable plasmids do not interfere in the analyses of astroglial clones since they are diluted through multiple progenitor cell divisions (García-Marqués and López-Mascaraque, 2012; Martín-López et al., 2013; García-Marqués et al., 2014). This approach, besides to permit the cell tracing, can be extrapolated to other genetic strategies applied to low vs. high dividing cells.

### *In vivo* cell lineage tracking depends on the electroporation stage

The expression of integrable vs. non-integrable constructs for the same cell lineage was different depending on electroporation stage. Therefore, it is important the electroporation time point correlated with the lineage commitment of the progenitor.

Our cell fate lineage analysis of OB interneurons revealed different results based on whether the electroporation was performed at embryonic or postnatal stages. SVZ produces neuroblasts that migrate to the OB and differentiate into interneurons throughout perinatal and postnatal life (Altman and Das, 1965; Lois and Alvarez-Buylla, 1994). We highlighted the requirement of the use of integrable constructs to target OB interneurons after IUE, since their OB progenitors undertake a large number of divisions before differentiating to that population (Miller and Gauthier, 2007; Kriegstein and Alvarez-Buylla, 2009). In fact, radial glia cells from the embryonic ventricular zone give rise to cortical cell types and intermediate progenitors before producing OB interneurons (Ming and Song, 2005). At postnatal ages, radial glia transforms into B-cells that first generate C-cells (intermediate progenitors) to finally differentiate into A-cells (neuroblasts) (Doetsch et al., 1997) accomplishing a huge amplification. Nevertheless, we detected that immature OB interneurons and RMS-neuroblasts co-expressed both integrable and non-integrable constructs 5 days after postnatal electroporation. It is not probable that those transfected cells were derived from a B-cell of the postnatal SVZ since lineage progression from B-cells to neuroblast takes in between 3 and 4 days (Ponti et al., 2013). Probably those cells came from the electroporation of advanced intermediate stages cells, such as C-cells or neuroblasts, with a reduced number of divisions to allow the dilution of non-integrate plasmid. Thus, electroporation age (embryonic or postnatal) was important since dilution of non-integrable constructs was related to the lineage-commitment of progenitors. In fact, when RMS- and OB-cells were analyzed 1 month after postnatal electroporation, the number of red cells (non-integrable plasmid) decreased in the RMS, indicating that new generated OB interneurons only expressed integrable plasmids. Indeed, the percentage of green (integrable construct) transfected OB interneurons highly increased at long-term analyses. Time-lapse studies of SVZ progenitors estimate that after the initial division of B1 cells, C cells divide three times and neuroblasts up to 2 times (Ponti et al., 2013). These data correlated with the lack of non-integrable construct in the RMS through time, since those neuroblasts came from SVZ progenitors that divided several times before attaining the neuronal fate. Other authors targeted OB interneurons after 21 days post-electroporation of non-integrable constructs (Fernández et al., 2011), while labeled SVZ- and RMS-cells were lost after 3–4 weeks of postnatal electroporation of non-integrable constructs (Lacar et al., 2010).

### *In vivo* lineage cell tracking ultimately depends on the cell identity

Our data indicated that glial cells were mostly green labeled carrying the integrable plasmid either after embryonic or postnatal electroporation. For instance, all the astrocytes expressed only the green construct regardless the stage of electroporation, which provides additional information about the embryonic division pattern of astrocyte-committed lineage progenitors (for review: Wang and Bordey, 2008). According to the postnatal astrocyte generation, precursors of those cells arrive to the final destination and actively proliferate there (Ge et al., 2012). Thus, it is correlated with the lost of non-integrated reporter, due to the elevate rate of cell divisions exhibited in this glial lineage.

Even though, we showed some glial cell populations, as NG2 and oligodendrocytes, which expressed the non-integrable construct, only the integrable reporter labeled the majority. Indeed, our recent data revealed that NG2 cells, form clones comprised by various hundreds of cells in adult brain (García-Marqués et al., 2014). The expression of non-integrated reporters after embryonic electroporation could be explained due to the existence of two main sources (ventral and cortical) of oligodendrocyte progenitors (Kessaris et al., 2006; Richardson et al., 2006). Dorsal electroporated oligodendrocyte-committed progenitors, originated in ventral regions, produce mature oligodendrocytes after fewer divisions than those from cortical regions. That would result in the permanence of the non-integrable reporters. On the other hand, when transfected cells are non-committed progenitors (e.g., radial glia or intermediate progenitors) the oligodendrocytes would differentiate after a larger number of divisions, resulting in the loss of the red non-integrable construct. Our approach will be helpful to address whether a different proliferative origin might be correlated with functional heterogeneity.

Remarkably, after postnatal electroporation of the dorso-lateral SVZ, transfected glial cells arranged in groups of a few cells, located throughout the gray matter (lower cortical layers and striatum) and white matter (corpus callosum). Those groups of cells, reduced in number, showed only the integrated green construct suggesting that they originated from amplified precursors, as intermediate progenitors. Furthermore, these electroporated progenitors give rise to interneurons and glial cells through multiple amplification of precursor, since most differentiate cells only expressed the green fluorescent protein. This suggests that the lineage determines the cell division pattern regardless of the progenitor stage, since glial cells are generated from multiple divisions, despite coming from postnatal glial lineage-committed progenitors.

### Co-electroporation of integrable and non-integrable plasmids as an indirect measure of proliferative potentials

Clonal studies with retroviral libraries (Levison et al., 1999; Zerlin et al., 2004) showed mixed glial clones originated from postnatal SVZ progenitors. Retroviruses are integrated into dividing cells at the injection site, providing information of the lineages that mitotic cells are going to produce, but not on other non-dividing progenitors lining the ventricle at the injection time. However, electroporation allows the tracking of the whole progeny of both quiescent and mitotic cells lining the ventricle at the electroporation time. Analyses of the SVZ, 30 days after postnatal electroporation, evidenced the presence of a huge number of SVZ labeled cells with a B cell morphology co-expressing both plasmids, since B-cells generated different cell types by asymmetric divisions (Rothenaigner et al., 2011). Probably, those cells remained un-dividing or “short-diving” within the SVZ as they highly expressed both fluorophores. Thus, those cells divided a limited number of times compared to the intermediate progenitors or neuroblasts, which is consistent with the presence of a large number of quiescent neural progenitors in the SVZ (Song et al., 2012).

All together, these results validate the comparison between integrated vs. non-integrated reporters as a method for an indirect measure of the proliferation rate. Comparative expression analyses after embryonic or postnatal co-electroporation in the cerebral cortex and in the SVZ-RMS-OB, showed that neuronal lineages arise from many progenitors in proliferative zones after few divisions. By contrast, glial lineages come from fewer progenitors that accomplish many cell divisions.

### Concluding remarks

The integration into the host genome of the reporter plasmids is required for a successful long-term lineage tracking of glial and OB interneurons generated from both dorso-lateral SVZ embryonic progenitors, as well as postnatal cell populations. Moreover, the use of integrable constructs allows the maintenance of cell labeling within the electroporated area, providing information about the origin of these differentiated cells, essential in ontogenetic and cell migration studies. The use of integrable and non-integrable plasmids is a valuable tool for further exploration of both quiescent cells and cell populations with a high division rate. Stable expression in both neuronal and glial lineages validates the method to track the dividing pattern potential for all brain areas and cell populations. Additionally, our data highlighted the importance of stable and persistent expression of non-integrable fluorescent reporters that remained in both cortical neurons generated at embryonic stages and throughout the postnatal SVZ-RMS-OB pathway. In addition, the comparison of differential expression of integrable and non-integrable plasmids might also be an indirect measure of the relative number of divisions before cell differentiation. Finally, the expression of non-integrated reporters vs. integrated allows studying the heterogeneity of neural populations originated from different proliferative rates.

### Conflict of interest statement

The authors declare that the research was conducted in the absence of any commercial or financial relationships that could be construed as a potential conflict of interest.
